# Dichloro­diphenoxy­methane

**DOI:** 10.1107/S160053680706789X

**Published:** 2008-01-04

**Authors:** Richard Betz, Peter Klüfers, Moritz M. Reichvilser

**Affiliations:** a Ludwig-Maximilians-Universität, Department Chemie und Biochemie, Butenandtstrasse 5–13, 81377 München, Germany

## Abstract

The title compound, C_13_H_10_Cl_2_O_2_, is a mixed derivative of orthocarbonic acid. The non-crystallographic symmetry of the mol­ecule is close to *C*_2*v*_. The aromatic residues are oriented in a *syn* conformation with respect to the Cl atoms. The least-squares planes through the phenyl rings enclose an angle of 36.11 (10)°. The C—O bonds at the central carbon are relatively short, and the O—C—O and Cl—C—Cl angles are smaller than the tetra­hedral angle. These metrical peculiarities including a mol­ecular symmetry close to *C*_2*v*_ are also observed in density functional theory (DFT) calculations, thus ruling out the decisive influence of inter­molecular forces in the crystal structure. Accordingly, only few and weak inter­molecular inter­actions are found. At distances smaller than the sum of the van der Waals radii, only two attractive inter­actions are detected: a weak C—H⋯O and a weak C—H⋯Cl hydrogen bond to one of the two potential acceptor atoms each.

## Related literature

For the synthesis of the title compound, see Bromley *et al.* (1996 ▶). For the crystal structure of related tetra­aryl­oxymethanes with slightly longer C—O bonds, see Narasimhamurthy *et al.* (1990 ▶).
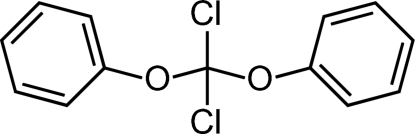


## Experimental

### 

#### Crystal data

C_13_H_10_Cl_2_O_2_
*M* *_r_* = 269.11
Monoclinic, 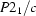

*a* = 15.8380 (4) Å
*b* = 5.8973 (2) Å
*c* = 14.2517 (4) Å
β = 114.751 (2)°
*V* = 1208.85 (6) Å^3^
*Z* = 4
Mo *K*α radiation
μ = 0.52 mm^−1^
*T* = 200 (2) K
0.22 × 0.20 × 0.15 mm

#### Data collection

Nonius KappaCCD diffractometer
Absorption correction: none
9193 measured reflections
2766 independent reflections
2138 reflections with *I* > 2σ(*I*)
*R*_int_ = 0.034

#### Refinement

*R*[*F* ^2^ > 2σ(*F* ^2^)] = 0.037
*wR*(*F* ^2^) = 0.094
*S* = 1.07
2766 reflections
154 parameters
H-atom parameters constrained
Δρ_max_ = 0.26 e Å^−3^
Δρ_min_ = −0.29 e Å^−3^

### 

Data collection: *COLLECT* (Nonius, 2004 ▶); cell refinement: *SCALEPACK* (Otwinowski & Minor, 1997 ▶); data reduction: *DENZO* and *SCALEPACK* (Otwinowski & Minor, 1997 ▶); program(s) used to solve structure: *SHELXS97* (Sheldrick, 1997 ▶); program(s) used to refine structure: *SHELXL97* (Sheldrick, 1997 ▶); molecular graphics: *ORTEPIII* (Burnett & Johnson, 1996 ▶); software used to prepare material for publication: *SHELXL97*.

## Supplementary Material

Crystal structure: contains datablocks I, global. DOI: 10.1107/S160053680706789X/hg2358sup1.cif

Structure factors: contains datablocks I. DOI: 10.1107/S160053680706789X/hg2358Isup2.hkl

Additional supplementary materials:  crystallographic information; 3D view; checkCIF report

## Figures and Tables

**Table:** 

O1—C1	1.359 (2)
O2—C1	1.360 (2)

**Table:** 

O1—C1—O2	103.77 (13)
Cl1—C1—Cl2	105.29 (9)

**Table 2:** Hydrogen-bond geometry (Å, °)

*D*—H⋯*A*	*D*—H	H⋯*A*	*D*⋯*A*	*D*—H⋯*A*
C3—H3⋯Cl1^i^	0.95	2.81	3.723 (2)	161
C10—H10⋯O1^ii^	0.95	2.52	3.345 (3)	145

Symmetry codes: (i) 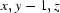; (ii) 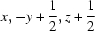.

**Fig. 1.**
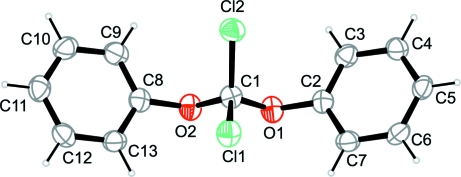
The molecular structure of (I), with atom labels and anisotropic displacement ellipsoids (drawn at 50% probability level) for non-H atoms.

**Fig. 2.**
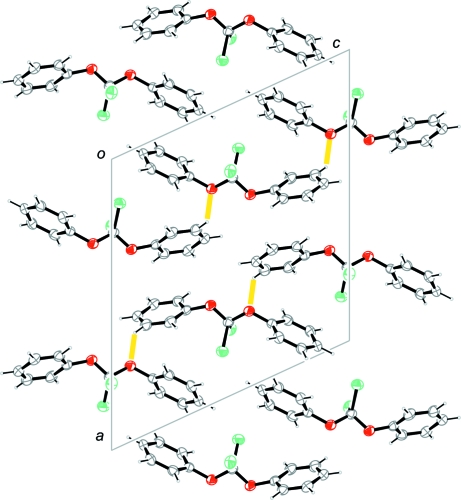
The packing of (I) viewed along [0 1 0], C—H···O hydrogen bonds drawn as yellow bars.

## supplementary crystallographic information

### Comment

The title compound (I) was prepared as starting material for the synthesis of spirocyclic orthocarbonates.

In the molecule the two aromatic moieties are oriented *syn* with respect to the Cl atoms (Fig. 1). The bond lengths between the central C atom and the O atoms are slightly shorter than in related tetraaryloxymethanes (Narasimhamurthy *et al.*, 1990). Unexpectedly, both the O—C—O and the Cl—C—Cl angles are smaller than the tetrahedral angle. The best planes through the phenyl moieties enclose an angle of 36.11 (10)°.

The molecular packing is shown in Figure 2. Below the limit of the sum of the van-der-Waals radii, one weak C—H···O and one weak C—H···Cl hydrogen bond were found in the asymmetric unit as well as an electrostatically repulsive C—H···H—C contact precisely at the vdW radii sum. No π stacking and no C—H···π contacts were observed within this cutoff criterion.

In agreement with the only weak intermolecular forces, the short bonds to the central carbon atom as well as the small bond angles mentioned above are corroborated by a DFT calculation on the B3LYP/6–311+G(2 d,p) level of theory.

### Experimental

The title compound was prepared according to a published procedure (Bromley *et al.*, 1996) upon chlorination of diphenylcarbonate with PCl_5_. Crystals suitable for X-ray analysis were obtained directly from the solid reaction product.

### Refinement

H atoms were refined as riding on their parent atoms with *U*_iso_ as the 1.2-fold of the pilot atom's *U*_eq_.

### Crystal data

**Table:**

C_13_H_10_Cl_2_O_2_	*F*_000_ = 552
*M**_r_* = 269.11	*D*_x_ = 1.479 Mg m^−^^3^
Monoclinic, *P*2_1_/*c*	Mo *K*α radiation λ = 0.71073 Å
Hall symbol: -P 2ybc	Cell parameters from 12368 reflections
*a* = 15.8380 (4) Å	θ = 3.1–27.5º
*b* = 5.8973 (2) Å	µ = 0.52 mm^−^^1^
*c* = 14.2517 (4) Å	*T* = 200 (2) K
β = 114.751 (2)º	Block, colourless
*V* = 1208.85 (6) Å^3^	0.22 × 0.20 × 0.15 mm
*Z* = 4	

### Data collection

**Table:**

Nonius KappaCCD diffractometer	2138 reflections with *I* > 2σ(*I*)
Radiation source: rotating anode	*R*_int_ = 0.034
Monochromator: MONTEL, graded multilayered X-ray optics	θ_max_ = 27.5º
*T* = 200(2) K	θ_min_ = 3.2º
CCD; rotation images; thick slices scans	*h* = −20→20
Absorption correction: none	*k* = −7→7
9193 measured reflections	*l* = −18→18
2766 independent reflections	

### Refinement

**Table:**

Refinement on *F*^2^	Secondary atom site location: difference Fourier map
Least-squares matrix: full	Hydrogen site location: inferred from neighbouring sites
*R*[*F*^2^ > 2σ(*F*^2^)] = 0.037	H-atom parameters constrained
*wR*(*F*^2^) = 0.094	*w* = 1/[σ^2^(*F*_o_^2^) + (0.0387*P*)^2^ + 0.3721*P*] where *P* = (*F*_o_^2^ + 2*F*_c_^2^)/3
*S* = 1.08	(Δ/σ)_max_ < 0.001
2766 reflections	Δρ_max_ = 0.26 e Å^−^^3^
154 parameters	Δρ_min_ = −0.29 e Å^−^^3^
Primary atom site location: structure-invariant direct methods	Extinction correction: none

### Fractional atomic coordinates and isotropic or equivalent isotropic displacement parameters (Å^2^)

**Table:**

	*x*	*y*	*z*	*U*_iso_*/*U*_eq_	
Cl1	0.22903 (3)	0.81935 (8)	−0.00008 (4)	0.04218 (16)	
Cl2	0.16437 (3)	0.38393 (9)	0.03397 (4)	0.04247 (16)	
O1	0.26019 (8)	0.4390 (2)	−0.07857 (9)	0.0370 (3)	
O2	0.34125 (8)	0.4761 (2)	0.08404 (9)	0.0367 (3)	
C1	0.25543 (12)	0.5198 (3)	0.00839 (13)	0.0327 (4)	
C2	0.18098 (12)	0.4454 (3)	−0.17458 (13)	0.0323 (4)	
C3	0.12316 (13)	0.2594 (3)	−0.20312 (14)	0.0367 (4)	
H3	0.1345	0.1336	−0.1578	0.044*	
C4	0.04817 (13)	0.2588 (4)	−0.29911 (14)	0.0379 (4)	
H4	0.0071	0.1327	−0.3198	0.046*	
C5	0.03320 (13)	0.4419 (3)	−0.36477 (14)	0.0371 (4)	
H5	−0.0187	0.4424	−0.4301	0.045*	
C6	0.09367 (13)	0.6242 (3)	−0.33543 (14)	0.0394 (5)	
H6	0.0836	0.7484	−0.3813	0.047*	
C7	0.16881 (13)	0.6273 (3)	−0.23968 (14)	0.0375 (4)	
H7	0.2108	0.7516	−0.2194	0.045*	
C8	0.36336 (11)	0.5446 (3)	0.18686 (14)	0.0332 (4)	
C9	0.34856 (13)	0.3947 (3)	0.25296 (15)	0.0404 (5)	
H9	0.3195	0.2525	0.2286	0.048*	
C10	0.37686 (13)	0.4557 (4)	0.35513 (16)	0.0448 (5)	
H10	0.3670	0.3547	0.4015	0.054*	
C11	0.41948 (12)	0.6623 (4)	0.39062 (15)	0.0418 (5)	
H11	0.4382	0.7039	0.4609	0.050*	
C12	0.43477 (13)	0.8085 (3)	0.32332 (15)	0.0411 (5)	
H12	0.4645	0.9500	0.3477	0.049*	
C13	0.40687 (12)	0.7493 (3)	0.22053 (15)	0.0377 (4)	
H13	0.4177	0.8486	0.1742	0.045*	

### Atomic displacement parameters (Å^2^)

**Table:**

	*U*^11^	*U*^22^	*U*^33^	*U*^12^	*U*^13^	*U*^23^
Cl1	0.0470 (3)	0.0337 (3)	0.0407 (3)	0.0093 (2)	0.0133 (2)	0.0033 (2)
Cl2	0.0378 (3)	0.0472 (3)	0.0451 (3)	−0.0041 (2)	0.0199 (2)	−0.0028 (2)
O1	0.0306 (7)	0.0447 (8)	0.0353 (7)	0.0066 (5)	0.0134 (5)	−0.0046 (6)
O2	0.0288 (6)	0.0427 (8)	0.0357 (7)	0.0091 (5)	0.0108 (5)	0.0002 (6)
C1	0.0302 (9)	0.0314 (10)	0.0352 (10)	0.0054 (7)	0.0126 (8)	0.0009 (8)
C2	0.0297 (9)	0.0371 (10)	0.0318 (9)	0.0045 (7)	0.0146 (7)	−0.0024 (8)
C3	0.0459 (11)	0.0300 (10)	0.0354 (10)	0.0030 (8)	0.0182 (9)	0.0028 (8)
C4	0.0406 (10)	0.0394 (11)	0.0361 (10)	−0.0066 (8)	0.0183 (8)	−0.0059 (9)
C5	0.0385 (10)	0.0471 (12)	0.0277 (9)	0.0000 (8)	0.0157 (8)	−0.0011 (9)
C6	0.0474 (11)	0.0417 (11)	0.0335 (10)	−0.0002 (9)	0.0213 (9)	0.0068 (9)
C7	0.0411 (10)	0.0368 (11)	0.0398 (10)	−0.0064 (8)	0.0222 (9)	−0.0028 (9)
C8	0.0253 (9)	0.0366 (10)	0.0341 (9)	0.0071 (7)	0.0090 (7)	0.0029 (8)
C9	0.0337 (10)	0.0350 (11)	0.0450 (11)	0.0009 (8)	0.0092 (8)	0.0085 (9)
C10	0.0366 (10)	0.0520 (13)	0.0420 (11)	0.0004 (9)	0.0128 (9)	0.0154 (10)
C11	0.0320 (10)	0.0557 (13)	0.0347 (10)	0.0041 (9)	0.0108 (8)	0.0024 (9)
C12	0.0319 (10)	0.0413 (12)	0.0428 (11)	−0.0037 (8)	0.0085 (8)	−0.0018 (9)
C13	0.0318 (10)	0.0407 (11)	0.0394 (11)	−0.0008 (8)	0.0136 (8)	0.0081 (9)

### Geometric parameters (Å, °)

**Table:**

Cl1—C1	1.8078 (18)	C6—C7	1.385 (3)
Cl2—C1	1.8154 (18)	C6—H6	0.9500
O1—C1	1.359 (2)	C7—H7	0.9500
O1—C2	1.418 (2)	C8—C13	1.373 (3)
O2—C1	1.360 (2)	C8—C9	1.381 (3)
O2—C8	1.415 (2)	C9—C10	1.380 (3)
C2—C3	1.377 (3)	C9—H9	0.9500
C2—C7	1.378 (3)	C10—C11	1.382 (3)
C3—C4	1.386 (3)	C10—H10	0.9500
C3—H3	0.9500	C11—C12	1.384 (3)
C4—C5	1.383 (3)	C11—H11	0.9500
C4—H4	0.9500	C12—C13	1.386 (3)
C5—C6	1.383 (3)	C12—H12	0.9500
C5—H5	0.9500	C13—H13	0.9500
			
C1—O1—C2	120.43 (13)	C7—C6—H6	119.7
C1—O2—C8	119.92 (13)	C2—C7—C6	118.23 (18)
O1—C1—O2	103.77 (13)	C2—C7—H7	120.9
O1—C1—Cl1	112.41 (12)	C6—C7—H7	120.9
O2—C1—Cl1	111.39 (13)	C13—C8—C9	121.85 (18)
O1—C1—Cl2	112.36 (12)	C13—C8—O2	118.89 (16)
O2—C1—Cl2	111.80 (12)	C9—C8—O2	118.98 (17)
Cl1—C1—Cl2	105.29 (9)	C10—C9—C8	118.69 (19)
C3—C2—C7	122.22 (17)	C10—C9—H9	120.7
C3—C2—O1	118.32 (16)	C8—C9—H9	120.7
C7—C2—O1	119.22 (16)	C9—C10—C11	120.60 (19)
C2—C3—C4	118.85 (18)	C9—C10—H10	119.7
C2—C3—H3	120.6	C11—C10—H10	119.7
C4—C3—H3	120.6	C10—C11—C12	119.71 (19)
C5—C4—C3	119.95 (18)	C10—C11—H11	120.1
C5—C4—H4	120.0	C12—C11—H11	120.1
C3—C4—H4	120.0	C11—C12—C13	120.33 (19)
C4—C5—C6	120.11 (18)	C11—C12—H12	119.8
C4—C5—H5	119.9	C13—C12—H12	119.8
C6—C5—H5	119.9	C8—C13—C12	118.81 (18)
C5—C6—C7	120.59 (18)	C8—C13—H13	120.6
C5—C6—H6	119.7	C12—C13—H13	120.6
			
C2—O1—C1—O2	177.16 (14)	C3—C2—C7—C6	2.3 (3)
C2—O1—C1—Cl1	−62.36 (19)	O1—C2—C7—C6	176.56 (16)
C2—O1—C1—Cl2	56.20 (19)	C5—C6—C7—C2	−0.6 (3)
C8—O2—C1—O1	178.00 (15)	C1—O2—C8—C13	−94.4 (2)
C8—O2—C1—Cl1	56.83 (19)	C1—O2—C8—C9	91.6 (2)
C8—O2—C1—Cl2	−60.67 (19)	C13—C8—C9—C10	1.3 (3)
C1—O1—C2—C3	−91.8 (2)	O2—C8—C9—C10	175.07 (16)
C1—O1—C2—C7	93.7 (2)	C8—C9—C10—C11	−0.2 (3)
C7—C2—C3—C4	−2.4 (3)	C9—C10—C11—C12	−0.7 (3)
O1—C2—C3—C4	−176.73 (15)	C10—C11—C12—C13	0.5 (3)
C2—C3—C4—C5	0.8 (3)	C9—C8—C13—C12	−1.4 (3)
C3—C4—C5—C6	0.9 (3)	O2—C8—C13—C12	−175.20 (16)
C4—C5—C6—C7	−1.0 (3)	C11—C12—C13—C8	0.5 (3)

### Hydrogen-bond geometry (Å, °)

**Table:**

*D*—H···*A*	*D*—H	H···*A*	*D*···*A*	*D*—H···*A*
C3—H3···Cl1^i^	0.95	2.81	3.723 (2)	161
C10—H10···O1^ii^	0.95	2.52	3.345 (3)	145

Symmetry codes: (i) *x*, *y*−1, *z*; (ii) *x*, −*y*+1/2, *z*+1/2.
